# Challenges in diagnosing and treating chronic exertional compartment syndrome: perspective from a student nurse with lower extremity pain

**DOI:** 10.3389/fphys.2025.1644047

**Published:** 2025-12-10

**Authors:** Madelyne Marbel, Robert Knoerl

**Affiliations:** Department of Health Behavior and Clinical Sciences, University of Michigan School of Nursing, Ann Arbor, MI, United States

**Keywords:** chronic exertional compartment syndrome, chronic pain, fasciotomy, intramuscular pressure monitoring, musculoskeletal pain

## Abstract

Chronic Exertional Compartment Syndrome (CECS) is an uncommon yet debilitating condition that causes activity-induced pain due to increased pressure within muscle compartments. While typically diagnosed through dynamic intercompartmental pressure testing, CECS presents significant diagnostic challenges due to overlapping symptoms with other musculoskeletal conditions. This first-person narrative describes a 23-month journey through inconclusive imaging, multiple rounds of physical therapy, and pressure testing that met, but was ultimately dismissed despite established CECS diagnostic criteria. The case underscores the mismatch that can occur between objective findings and clinical interpretation, particularly when symptoms affect atypical compartments. With surgery ruled out and conservative treatments offering minimal relief, the experience reveals critical gaps in diagnostic consistency, clinician-patient communication, and care pathways for individuals whose symptoms reside in a diagnostic gray zone. This narrative calls for more nuanced application of diagnostic tools, greater attention to patient-reported symptoms, and improved support systems for those navigating prolonged diagnostic uncertainty.

## Introduction

Chronic Exertional Compartment Syndrome (CECS) is a relatively rare condition that is characterized by exercise-induced pain, cramping, tightness, paresthesia and/or muscle weakness in the upper or lower extremities (Tarabishi et al., 2023). The symptoms generally subside once exercise or extensive activity cease (Ding et al., 2020). CECS is commonly found in athletes (e.g., runners, soccer players) or military personnel members with high levels of repetitive activity (Velasco and Leggit, 2020). Specifically, some data suggest that approximately half of individuals who report lower extremity leg-pain and undergo compartment pressure testing are diagnosed with CECS (Davis et al., 2013; de Bruijn et al., 2018). It is believed that CECS arises from increased intra-compartmental pressures within inelastic fascial compartments during exercise. These pressures subsequently restrict blood flow to the muscles and surrounding tissue, resulting in pain and dysfunction (Tarabishi et al., 2023; Velasco and Leggit, 2020). The assessment and subsequent diagnosis of CECS is challenging due to symptom overlap with other conditions (e.g., stress factures or medial tibial stress syndrome) (Velasco and Leggit, 2020). Delayed diagnosis of CECS can lead to persistent chronic pain that reduces physical activity (e.g., activities of daily living or stop playing sports) and overall quality of life (e.g., worsened mental health) (Nwakibu et al., 2020).

What follows is not a definitive claim of having CECS, but a personal account as a patient and nursing student of living with objective findings that meet CECS criteria and the treatment and diagnostic barriers I faced. My story highlights the clinical and emotional consequences of diagnostic uncertainty surrounding CECS diagnosis and treatment. I share this story to shed light on the patient perspective and to urge healthcare providers to better recognize, support, and advocate for those navigating diagnostic limbo.

## 23 Months to failure

In strength training, “training to failure” refers to pushing the body to its limit (e.g., continuing until it can no longer maintain proper form). When done intentionally, it can foster growth. But living in a constant state of failure (e.g., marked by pain and medical uncertainty) is another story entirely. For 23 months, I lived in that space: enduring pain, navigating a rotating cast of providers, and chasing a diagnosis that was and remains elusive.

## Diagnostic ambiguity and reliance on exclusion

My athletic history mirrors the typical CECS profile. I was a dedicated runner from a young age, completed my first triathlon at 13, and competed in track and field through high school. This lifestyle of sustained physical intensity may have made me more vulnerable to CECS-like symptoms. In February 2023, I completed what I did not realize would be the most pivotal 7-mile run of my life. Shortly afterward, I began experiencing burning, exercise-induced pain in my lower leg, symptoms triggered not by running, but by everyday tasks like walking to class. Soon, even gentle inclines brought searing pain within minutes, regardless of pace. What initially seemed like overtraining evolved into something no one could clearly diagnose.

CECS is largely a diagnosis of exclusion (Velasco and Leggit, 2020) with no universally accepted criteria for confirmation (Tarabishi et al., 2023). As a result, patients may undergo a comprehensive health history and a battery of tests to rule out more common causes of exercise-induced lower extremity pain (Velasco and Leggit, 2020; der Kraats et al., 2023). Common non-invasive diagnostic tools include magnetic resonance imaging (MRI), near-infrared spectroscopy, electromyography, single-photon emission computer tomography, and ultrasound, but there is not strong data supporting the use of these tools for diagnosing CECS (der Kraats et al., 2023). I had MRI tests to rule out possible muscle tears or strains of the affected compartment, X-rays to assess for bone abnormalities, and lab work to exclude systemic or vascular issues. Other diagnoses to rule out before a CECS diagnosis may be confirmed include stress fractures, shin splints, or nerve entrapment (Tarabishi et al., 2023). Each inconclusive test brought a wave of hope followed by disappointment.

The diagnoses related to my lower extremity pain started with muscle tightness, for which I was prescribed stretching by my first physical therapist. That diagnosis progressed into patellofemoral syndrome, or “runner’s knee.” I did not have a clinical background in kinesiology, but I became increasingly attuned to my body’s biomechanics. As inflammation restricted my lower leg, I believe my altered gait overloaded my knee, leading to joint maltracking. Ironically, the prescribed stretches worsened my symptoms, leaving my muscles tighter and my mobility more limited. I continued the regimen, hoping consistency would eventually bring relief, but I never imagined that nearly 2 years later, I would still be searching for answers.

## Atypical symptom presentation

After 18 months of persistent lower extremity leg pain and no improvement in pain from conservative treatments like physical therapy, my presentation no longer fit the classic definition of CECS (e.g., dull, achy, cramping pain upon activity) (Vajapey and Miller, 2017). Instead of pain that subsided immediately or within minutes after stopping activity, the pain lingered long afterward. By October 2024, I could no longer walk to my nursing classes. Bearing weight on my leg triggered searing, relentless pain. It was not until my fifth physical therapy clinic, nearly 20 months in, that I met a provider who truly listened. Under her care, I tried strengthening exercises, dry needling, laser therapy, and nerve glides to relieve muscle tension from disuse, but nothing helped. I focused on rebuilding larger muscle groups to offload smaller, overburdened ones. Still, I could not shake the feeling that something deeper had shifted since that February run.

One issue that delayed my diagnostic process was that I was not experiencing pain in the anterior or lateral compartments (i.e., where CECS is most commonly diagnosed) (der Kraats et al., 2023). As a result, some clinicians began to discount CECS as a diagnosis. The first physical medicine and rehabilitation (PM&R) specialist I saw told me definitively that I did not have CECS solely because the “wrong” compartment was affected. At the time, I reported most of my pain in the medial compartment.

## Dismissal and borderline findings

Seeking further answers, I pursued a second opinion in my hometown, where a physician referred me for dynamic intercompartmental pressure (IMP) testing (i.e., considered the gold standard for diagnosing CECS) (der Kraats et al., 2023; Pedowitz et al., 1990). The procedure was both physically and emotionally taxing. After prepping, a thin-gauge needle attached to a pressure monitor was inserted into four leg compartments: anterior, lateral, superficial posterior, and deep posterior. Readings were taken at rest, and again one and 5 minutes after symptom-provoking exercise (e.g., repeated calf raises). According to guidelines, CECS is present when pressures exceed 15 mmHg at rest, 30 mmHg 1 minute post-exercise, or 20 mmHg 5 minutes post-exercise (Pedowitz et al., 1990).

My results were inconclusive: 30 mmHg 1 minute post-exercise in the lateral compartment, and 25–26 mmHg 5 minutes post-exercise in the lateral and superficial posterior compartments. These met Pedowitz’s diagnostic thresholds for CECS, but the physician questioned their validity, attributing the elevations to an “involuntary increase in muscle tone” potentially caused by saline infiltration during the test. This interpretation rendered the findings unreliable in his view. Dismissing elevated readings due to an inherent aspect of the procedure itself calls into question how CECS is diagnosed in clinical practice.

Moreover, while I experienced pain in the medial compartment, the elevated pressures were recorded in the lateral and superficial posterior compartments. This disconnect between symptoms and test results echoes findings in the literature that pressure readings do not always correlate with patient experience (Roberts and Franklyn-Miller, 2012). For me, the test did not bring clarity but deepened the diagnostic uncertainty and left me without answers, a treatment plan, or relief from pain.

## After the test: managing pain without surgical options

Despite severe symptoms, my pressure readings did not meet the criteria for surgical intervention. Fasciotomy, the surgical release of the fascia, is considered the first-line treatment for CECS, and has shown greater efficacy than conservative strategies in reducing pain (Elsenosy et al., 2024). However, it carries risks such as infection and nerve injury (Nwakibu et al., 2020), and its long-term effectiveness remains debated (Thein et al., 2019). With surgery off the table, for financial reasons, I continued with self-directed physical therapy exercises I had been previously assigned. Conservative treatments like massage, rest, and stretching exist, but evidence supporting their effectiveness is limited (Nwakibu et al., 2020). My self-directed rehab became a trial-and-error process. Still, it helped me better understand my own biomechanics. The bigger issue was the lack of structured guidance. For patients like me (i.e., those with borderline test results but debilitating symptoms), there is no clear path forward. No protocol. No roadmap. The burden of navigating that uncertainty fell squarely on my shoulders.

## Discussion

My experience with suspected CECS illustrates the diagnostic and treatment challenges many patients face, particularly those whose symptoms do not conform neatly to textbook definitions or whose test results fall into borderline zones. Despite meeting established diagnostic thresholds for CECS in certain muscle compartments, my case was ultimately dismissed due to ambiguous interpretation and compartment location. This reflects a broader tension in medicine: the reliance on imperfect diagnostic tools to make definitive clinical decisions in the face of patient-reported suffering.

My experience underscores the limitations of relying heavily on dynamic intercompartmental pressure testing as the gold standard diagnostic tool. While IMP testing provides an objective measure, the validity of the diagnostic criteria for CECS has been called into question in various studies (Roberts and Franklyn-Miller, 2012; Roscoe et al., 2015; Simpson et al., 2020). As such, research has suggested new criteria for diagnosing CECS that builds upon the Pedowitz criteria (Roscoe et al., 2015). Diagnostic ambiguities can lead to dismissal of patient concerns, leaving patients in a state of “diagnostic limbo.” Clinicians should consider the patients’ symptom history rather than strict reliance on IMP thresholds.

Given the risks and uncertainties surrounding fasciotomy, conservative treatments remain the default for many patients with borderline or inconclusive findings, despite limited evidence supporting their efficacy (Nwakibu et al., 2020). This highlights a pressing need for more structured, evidence-based protocols to guide management in these cases, as well as research into novel diagnostic methods and therapeutic options. For example, cognitive behavioral therapy is a recommended treatment for chronic pain (Ehde et al., 2014), but less is known about the efficacy of cognitive behavioral therapy for chronic pain conditions like CECS.

Despite my training as a nurse and experience working alongside other healthcare professionals, I did not realize just how challenging it would be to have your concerns addressed when you are on the receiving end of care. This experience revealed that clinician-patient communication is an integral part of managing patient care. At every appointment, I felt an unseen barrier separating me from the very team I would have been part of if the circumstances were different, and yet those circumstances should not have changed anything. This experience revealed not only how easily patients can be dismissed but the systemic gaps in communication that continue to persist in healthcare. We must listen more deeply, validate more often, and follow up more intentionally when symptoms do not align neatly with diagnostic criteria. Further research is needed to explore patients’ perceptions regarding the diagnosis and management of CECS in rigorous qualitative studies.

## Conclusion

Ultimately, this case invites broader reflection on how we approach “gray area” diagnoses in musculoskeletal medicine. When objective findings are inconclusive but patient suffering is evident, we must ask whether our threshold for action is guided more by rigid metrics than by clinical judgment. A more holistic, patient-centered approach is needed—one that validates experience, bridges the gaps between subjective symptoms and objective tests, and offers compassionate care even when answers remain uncertain.

## Data Availability

The original contributions presented in the study are included in the article/supplementary material, further inquiries can be directed to the corresponding author.
